# Editorial: The Use of Routine Health Data in Low- and Middle-Income Countries

**DOI:** 10.3389/fpubh.2020.00413

**Published:** 2020-08-20

**Authors:** Jim Todd, Michael Johnson Mahande

**Affiliations:** ^1^London School of Hygiene and Tropical Medicine, University of London, London, United Kingdom; ^2^Kilimanjaro Christian Medical University College, Kilimanjaro, Tanzania

**Keywords:** data, public health, LMIC = low- and middle-income countries, Africa, health information, routine data

In most high income countries, routinely collected health data are regularly used to inform policy, and provide real-time updates of health concerns. Techniques and applications have been developed for a wide range of data, both aggregated data and for individual patient data. However, in low and middle income countries (LMIC) routinely collected data have not been used quite as much, partly because the data are not so easily available, and partly due to the dearth of data professionals to analyse the data (1). There have been several initiatives to increase the numbers of data professionals in sub-Saharan Africa (SSA), and to improve their skills. This topic aimed to explore the extent to which routine data for health are being used across LMIC and how this was being led by African researchers.

One area where the analysis of routine health has produced beneficial results is through electronic health records (EHR). In SSA there has been a lot of investment in EHR for HIV services, and we anticipated papers that used anonymized records of people living with HIV (PLHIV) and the impact of HIV treatment on survival and quality of life (2). Two papers in this collection identified ways to model HIV disease progression using routinely collected data from HIV clinics. From Ethiopia a Poisson-Gamma-Normal model was used to account for overdispersion of the data and correlation within subjects, while from Zimbabwe multistate Markov models were used to identify factors that were associated with disease progression (Andualem and Ayele; Matsena Zingoni et al.). Two other papers, both from Tanzania, used the HIV EHR to look at TB infections, with one paper reporting that 96% of clinic visits by PLHIV included screening for TB, and the other giving the incidence of TB among PLHIV as 2 per 1000 person-years (Mollel et al.). This experience demonstrate that routinely collected, openly-accessible health data from many countries promotes a wealth of analyses and papers, which feed into the wider picture for the impact of improved HIV services.

A different use for routine data came from the development of tools to get more accurate estimates of disease. In many countries of Africa, there is a dependence on surveys to provide measures of prevalence and incidence of disease (3). However, smaller scale estimates are needed to inform health services for communities and routine data from health facilities can be used to obtain those hybrid estimates (4). A paper from South Africa used spatial interpolation to develop tools to bring routinely collected health data in order to provide more accurate estimates of HIV prevalence at national and sub-national levels (Wabiri et al.). This is an inspiration to others working the field to generate new methods to anneal data in this way.

Another use of routine data is to improve health services. This can be done by using the data to show areas where more effort is needed, and by comparing different services using the data they generate. Several papers in this topic showed how health services can be improved through judicious use of the data they generate every month. This ranged from ways to improve the linkage between HIV testing and HIV care (Harklerode et al.) to highlighting the misreporting of patient outcomes if those to follow up are not properly accounted for (Etoori et al.). Routine health has its problems in assessing the impact of interventions in hospitals, not least because of missing data that has not been properly recorded. Gachau et al. showed that the use of missing data methods need to account for the hierarchical nature of the data, in order to correctly assess the impact of an audit and feedback intervention.

Health services for infants are of particular concern as there is often a need to link health records of children with those of their mother. In Tanzania, PATH are starting with immunization records to build the requirement for an electronic immunization registry (EIR), showing this needs to operate on different software platforms (Seymour et al.). In Zambia an increasing number of HIV-infected pregnant women are already on antiretroviral therapy (ART) when they first attend antenatal care (Gumede-Moyo et al.). SmartCare data in Zambia were used to assess the impact of the Option B+ program on subsequent HIV transmission and the impact of ART on the survival of children born with HIV infection (Muyunda et al.; Munthali et al.).

The importance of advanced statistical methods was evident in all the papers. One paper used survival techniques to study marital formation and dissolution in South Africa (Batidzirai et al.). This showed that with advanced statistical methods you can get a more accurate picture of the real risks for life events such as marital formation and dissolution. Another paper showed that time-varying covariates are needed to properly understand the survival of patients given a kidney transplant (Achilonu et al.).

All papers used some interesting advanced statistical methods, These ranged from Bayesian methods to model disease progression, through clustering of effects in hierarchical data, time-varying covariates in survival analyses, and adjusting for missing data. The application of these methods in LMIC, and in sub-Saharan Africa in particular, are crucial to the future analyses of routinely collected health data. These papers represent the first steps of African statisticians to come to grips with the complexity of analysis, and the exploration of Big Data related to the health of the African continent.

## Author Contributions

JT conceived the topic and provided editorial inputs to papers. MM supported the topic and provided editorial inputs to a number of papers. Together we identified the need to highlight analysis of routinely collected health data in many African countries.

## Conflict of Interest

The authors declare that the research was conducted in the absence of any commercial or financial relationships that could be construed as a potential conflict of interest.
